# The Plant Cuticle: An Ancient Guardian Barrier Set Against Long-Standing Rivals

**DOI:** 10.3389/fpls.2021.663165

**Published:** 2021-06-25

**Authors:** Gulab Chand Arya, Sutanni Sarkar, Ekaterina Manasherova, Asaph Aharoni, Hagai Cohen

**Affiliations:** ^1^Department of Vegetable and Field Crops, Institute of Plant Sciences, Agricultural Research Organization (ARO), Volcani Center, Rishon LeZion, Israel; ^2^Plant Pathology and Microbiology Department, Robert H. Smith Faculty of Agriculture, Food and Environment, The Hebrew University of Jerusalem, Rehovot, Israel; ^3^Department of Plant and Environmental Sciences, Weizmann Institute of Science, Rehovot, Israel

**Keywords:** plant cuticle, pathogenic fungi, defense response, epicuticular wax, plant-pathogen interactions

## Abstract

The aerial surfaces of plants are covered by a protective barrier formed by the cutin polyester and waxes, collectively referred to as the cuticle. Plant cuticles prevent the loss of water, regulate transpiration, and facilitate the transport of gases and solutes. As the cuticle covers the outermost epidermal cell layer, it also acts as the first line of defense against environmental cues and biotic stresses triggered by a large array of pathogens and pests, such as fungi, bacteria, and insects. Numerous studies highlight the cuticle interface as the site of complex molecular interactions between plants and pathogens. Here, we outline the multidimensional roles of cuticle-derived components, namely, epicuticular waxes and cutin monomers, during plant interactions with pathogenic fungi. We describe how certain wax components affect various pre-penetration and infection processes of fungi with different lifestyles, and then shift our focus to the roles played by the cutin monomers that are released from the cuticle owing to the activity of fungal cutinases during the early stages of infection. We discuss how cutin monomers can activate fungal cutinases and initiate the formation of infection organs, the significant impacts of cuticle defects on the nature of plant–fungal interactions, along with the possible mechanisms raised thus far in the debate on how host plants perceive cutin monomers and/or cuticle defects to elicit defense responses.

## Introduction

The aerial surfaces of plants are covered by a lipophilic protective shield called the cuticle. The cuticle acts as a diffusion barrier and, therefore, influences the diffusion of an array of molecules such as water, gases, and solutes (Isaacson et al., 2009; Chen et al., 2011). Yet, apart from enabling plants to survive in dry environments, the cuticle represents the first line of defense against biotic stresses triggered by a variety of pathogens and pests, including fungi, bacteria, and insects. Thus, the cuticle acts as the interface where the complex molecular interactions occur between plant surfaces and pathogens. Not surprisingly, many attributes of the cuticle, for example, its architecture, thickness, and biochemistry were associated with altered resistance or susceptibility to pathogens (Manandhar and Hartman, 1995; Gabler et al., 2003; Gomes et al., 2012; Martin and Rose, 2014).

**Figure 1:**
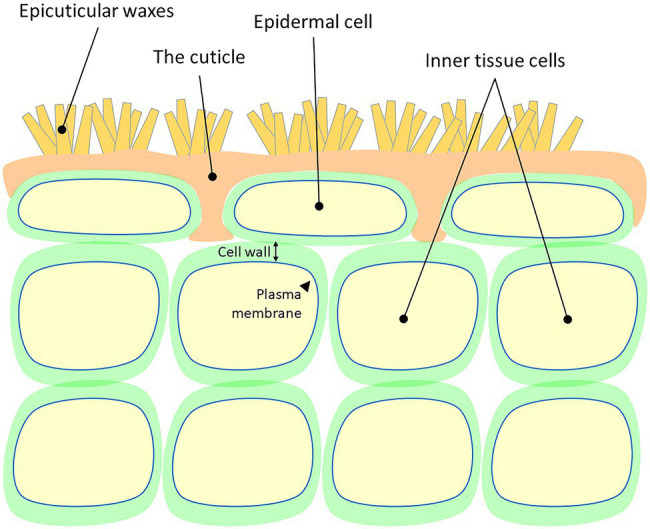
A schematic representation of the cellular localization of the plant cuticle. Cell wall and plasma membrane are presented.

Plant cuticles are made of lipophilic compounds that are deposited onto the outer cell walls of the epidermis layer (Figure 1). These include the solvent-extractable cuticular waxes and cutin, cuticle’s main component, which cannot be extracted due to its polymeric nature. Cuticular waxes are typically deposited within (intracuticular) or on top (epicuticular) of the cutin matrix and are composed of a mixture of C_20_ to C_40_ very-long-chain-fatty-acids (VLCFAs), which are further modified to form corresponding alkanes, aldehydes, ketones, primary and secondary alcohols, and esters (Samuels et al., 2008; Buschhaus and Jetter, 2011). The polyester cutin is composed of C_16_ and C_18_ fatty acids modified with functional groups, such as terminal and mid-chain hydroxy, epoxy, and carboxy groups, which are cross-linked by ester bonds (Cohen et al., 2019; Philippe et al., 2020). Studies show that the structural and chemical nature of the cuticle varies greatly between plant species, genotypes, organs, and developmental stages (Jeffree, 2006; Domínguez et al., 2011; Yeats and Rose, 2013; Fernández et al., 2016; Jetter and Riederer, 2016).

**Figure 2:**
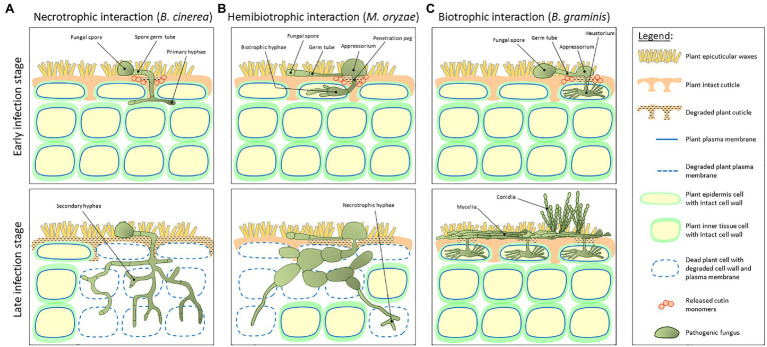
A schematic representation of the host plant and pathogen fundamental structures during early and late stages of plant–fungal interaction. **(A)** Interaction with a necrotrophic fungus (e.g., *Botrytis cinerea*). Spores land on the host plant cuticle surface and generate a germ tube. These tubes become the primary hyphae that penetrate through the cuticle and grow below the cuticle into a complex secondary hyphae structure that kills epidermal and inner tissue host cells. **(B)** Interaction with a hemibiotrophic fungus (e.g., *Magnaporthe oryzae*). At early stages of infection, spores germinate on the cuticle surface and develop a specialized infection structure called appressorium, a flattened organ that pressures the host plant surface eventually penetrating it *via* a penetration peg. This stage is considered biotrophic as the bulged hyphae that colonize the infected cells do not kill it. However, at later infection stages, these hyphae adopt a necrotrophic life style. **(C)** Interaction with a biotrophic fungus (e.g., *Blumeria graminis*). At early infection stage, spores germinate on the cuticle surface and develop an appressorium. After penetration through the host plant cuticle, the fungus colonizes the intercellular space *via* a feeding structure called haustorium, which invades the host cell without piercing the plasma membrane and killing it. At final stages of infection, the fungus produces dense mycelia on the cuticle surface and conidia. Upper panels represent early infection stages, whereas bottom panels represent late infection stages.

The development of the cuticle facilitated the terrestrialization of land plants approximately 450 million years ago (Cohen et al., 2017). As most primary nutritional source of carbon for fungal species is living or dead plant tissue, it is hypothesized that the early colonizing plants paved the way to the foundation and divergence of the first fungal ancestries (Lutzoni et al., 2018). Indeed, the main concept opines that plants and fungi coevolved 400–600 million years ago (Heckman et al., 2001), and that during this period, these two kingdoms developed complex relationships. These include symbiotic interactions where both the host plant and the fungus benefit from their mutualistic relationship; saprotrophy, where the fungus obtains nourishment from dead or decaying plant tissues; and parasitism, practiced by most pathogenic fungi, which need to penetrate the host plant tissue in order to reach the nutritional contents of inner cells (Burdon and Thrall, 2009). Plant colonization by fungi strongly depends on their lifestyle. Spores of necrotrophic fungal species (e.g., *Botrytis cinerea*) land on the host plant cuticle surface, germinate *via* germ tubes that eventually become the primary hyphae that penetrate through the host cuticle (Figure 2A, upper panel). Following penetration, hyphae grow below the cuticle to some complex secondary hyphae that kills epidermal and inner tissue host cells (Figure 2A, bottom panel). At the early stages of infection, hemibiotrophic fungal (e.g., *Magnaporthe oryzae*) spores germinate on the cuticle surface and develop a specialized infection structure called appressorium, a flattened organ that pressures the host plant surface eventually penetrating it *via* a penetration peg. This stage is considered biotrophic as the bulged hyphae that colonize the infected cells do not kill it (Figure 2B, upper panel). However, at later infection stages, these hyphae adopt a necrotrophic lifestyle eventually killing epidermal and inner tissue host cells (Figure 2B, bottom panel). Biotrophic fungi (e.g., *Blumeria graminis*) germinate on the cuticle surface and typically develop an appressorium. This structure penetrates through the host plant cuticle and colonizes the intercellular space *via* a feeding structure called haustorium, which invades the host cell without piercing the plasma membrane and killing it (Figure 2C, upper panel). At the final stages of infection, the fungus produces dense mycelia on the cuticle surface and conidia (Figure 2C, bottom panel; Schulze-Lefert and Panstruga, 2003; Laluk and Mengiste, 2010; Lo-Presti et al., 2015).

Pathogenic fungi have established a battery of strategies to overcome the cuticle barrier. These include the utilization of cuticle-derived signals that induce the spore germination on the plant surface, the formation of specialized infection organs, and penetration of the cuticle. Some fungal species enter through stomata or natural gaps, whereas others pierce the surface of the cuticle by applying mechanical pressure. Most pathogenic fungi, however, secrete a blend of specialized cell wall-degrading enzymes toward the plant surface, including pectate lyases, cellulases, and cutinases. The latter family of enzymes possesses a unique ability to release the ester bond-linked monomers that build the cutin polyester (Kubicek et al., 2014). Cutinase activity was shown in various pathogenic fungi to greatly impact the process of infection: from the initial stages of spore adhesion to the plant surface, through spore germination and the formation of specialized infection organs, to the breakdown of the cuticle and the colonization of the host plant (Kolattukudy, 1985).

Regardless of the kind of relationship, all the types of interaction between the aerial organs of plants and fungi take place at the cuticle surface – a hub of plant innate immunity and fungal infection responses. In the current review, we focus on the multidimensional roles of plant cuticle-derived components during the infection of pathogenic fungi. For additional information about the regulation of plant–bacteria interactions at the cuticle surface, we warmly refer readers to the excellent reviews of Aragón et al. (2017) and Ziv et al. (2018). Here, we first describe rudimentary evidence that establishes epicuticular waxes as major determinants of plant–fungal interactions and that certain wax components can affect various pre-penetration and infection processes of fungi with different lifestyles. We then shift our focus to the roles played by cutin monomers that are released from the cuticle owing to the activity of fungal cutinases during the early stages of infection. We describe how these monomers activate fungal cutinases and initiate the formation of infection organs. Finally, we mention key reports that revealed the significant impacts of imperfections in cuticle biochemistry and permeability on the nature of interactions between pathogenic fungi and host plants and discuss possible mechanisms by which host plants perceive released cutin monomers to elicit defense responses.

### Epicuticular Waxes Are Major Determining Factors of Plant–Fungal Interactions

As epicuticular waxes are deposited on top of the outermost surface of the cuticle, they are the first to interact with any type of pathogen. It is, therefore, expected that changes to the patterns of crystallization, composition, and hydrophobicity of epicuticular waxes will significantly impact various aspects of plant–fungal interactions (Shepherd and Griffiths, 2006; Buschhaus and Jetter, 2011; Lewandowska et al., 2020). Extensive work on *B. graminis* has shown that this pathogenic fungus exploits components of the plant epicuticular wax to induce pre-penetration processes. For instance, silencing 3-ketoacyl-CoA synthase 6 (KCS6) and enoyl-CoA reductase (ECR) in wheat (*Triticum aestivum*), both of which are important for VLCFA biosynthesis and the elongation reactions required for cuticular lipid biosynthesis, attenuates *B. graminis* spore germination (Wang et al., 2019; Kong et al., 2020). In line with these findings, Feng et al. (2009) characterized Lip1, a lipase in *B. graminis* that is secreted onto the surface of fungal cell walls and possesses the ability to release alkanes and primary fatty alcohols from the epicuticular wax of wheat leaves. Remarkably, the pretreatment of wheat leaves with Lip1 resulted in the removal of surface wax, which, in turn, severely compromised conidial adhesion, appressorium formation, and secondary hyphal growth of the fungus (Feng et al., 2009). The spores of this fungus also hardly germinated on the barley (*Hordeum vulgare*) *emr1* mutant, which is depleted in the leaf surface waxes due to a mutation in KCS6 (Weidenbach et al., 2014). In the case of *Alternaria brassicicola*, it was shown that the removal of epicuticular waxes from cauliflower (*Brassica oleracea*) leaves affected spore adhesion and fungal penetration during the early stages of infection (Berto et al., 1999). An intriguing case that highlights the divergent effects of the wax composition on fungal infection is that of *Curvularia eragrostidis*, a cosmopolitan fungal pathogen that infects hosts from several botanical families (Ferreira et al., 2014). It was found that epicuticular waxes from its grass host plant, hairy crabgrass (*Digitaria sanguinalis*), significantly induced spore germination and germ tube elongation, but had no effect on appressorium differentiation. Yet, the epicuticular waxes of tall fescue grass (*Festuca arundinacea*), which represents a nonhost species of this fungus, hindered fungal spore germination and appressoria formation (Wang et al., 2008).

The aforementioned studies clearly demarcate the importance of the signaling roles of epicuticular wax components to the initiation of fungal infection processes. Complementing investigations sought to isolate the specific wax components that initiate these processes. *In vitro* assays have validated that very-long-chain (VLC) aldehydes trigger the spore germination and appressorium differentiation of *B. graminis* (Ringelmann et al., 2009; Hansjakob et al., 2010, 2011). Similarly, wax inducer 1 (WIN1) suppression in wheat negatively affected *B. graminis* germination by interfering with the VLC aldehyde wax biosynthesis. Remarkably, coating the leaves of WIN1-silenced lines with typical wild-type (WT) epicuticular waxes fully restored the spore germination of the fungus (Kong and Chang, 2018). More specifically, the C_28_ aldehyde of wheat was shown to endorse the spore germination in *Puccinia graminis* f. sp. *tritici* (Reisige et al., 2006). Apart from the wax aldehydes, primary alcohols were also shown to play critical roles in the fungal infection initiation. Namely, the C_24_ primary alcohol on the surface of avocado (*Persea americana*) fruit triggers the spore germination and appressorium differentiation in *Colletotrichum gloeosporioides* (Podila et al., 1993). Higher levels of the C_30_ primary alcohol in the Arabidopsis *cer1* line, which is mutated in the ECERIFERUM 1 (CER1) enzyme, were able to suppress the growth and reproduction of *Golovinomyces orontii* (Jenks et al., 1995; Inada and Savory, 2011). Interestingly, the overexpression of CER1 in Arabidopsis promoted the VLC alkane biosynthesis, resulting in higher susceptibility to the infection by *Sclerotinia sclerotiorum* (Bourdenx et al., 2011), highlighting that the wax alkanes also play significant signaling roles. Correspondingly, the transgenic cucumber (*Cucumis sativus*) fruit with lower expression of CsWAX2, a homolog of the Arabidopsis WAX2 gene involved in the biosynthesis of VLC wax alkanes, resulted in an overall reduction of 50% in the wax content of the fruit surface, owing to major reductions in C_29_ and C_31_ alkanes. Inoculation assays with *B. cinerea* demonstrated that its pathogenicity was dramatically impaired only at the fruit surface of this mutant, but not on the fruit surfaces of WT and WAX2-overexpression plants (Wang et al., 2015). WAX2 was shown to be allelic to *CER3/YORE-YORE (YRE)/FACELESS POLLEN1* (*FLP1*) and has a pleiotropic phenotype, including an altered wax composition (Rowland et al., 2007). Finally, polar wax-associated terpenoids on the avocado fruit surface were shown to induce appressorium formation (Kolattukudy et al., 1995).

The findings described above emphasize that different epicuticular wax components can affect various processes of pathogenic fungal infection, yet these effects are seemingly far more complex than assumed, as demonstrated by Uppalapati et al. (2012). The forward-genetics screen using Barrel clover (*Medicago truncatula*) *Tnt1* retrotransposon insertion lines followed by the authors found that the *inhibitor of rust germ tube differentiation 1* (*irg1*) mutant failed to promote the pre-infection structural differentiation of two rust pathogens, *Phakopsora pachyrhizi* and *Puccinia emaculata*, on the abaxial leaf surface. The chemical analysis of the epicuticular wax composition revealed a >90% reduction in C_30_ primary alcohols and overaccumulation of C_29_ and C_31_ alkanes in the leaves of the *irg1* mutant (Uppalapati et al., 2012). Further analyses validated that IRG1 encodes the Cys(2)His(2) zinc transcription factor, PALM1, which plays important role in regulating epicuticular wax metabolism and transport. Yet, the most intriguing observation was that the altered wax composition in leaves of this mutant had entirely dissimilar effects on the virulence of pathogenic fungi with different lifestyles. As mentioned above, the *irg1* mutant inhibited the pre-infection structural differentiation of the rust fungal species *Phakopsora pachyrhizi* and *Puccinia emaculata*, but had no effect on the pathogenicity of the necrotrophic fungus *Phoma medicaginis* (Uppalapati et al., 2012), suggesting that the changes in leaf wax composition might be limited to fungal species that form pre-infection appressoria structures in response to the surface signals, unlike *Phoma medicaginis*, which directly penetrates the cuticle without forming these structures.

### Cutin Monomers Released During Infection Activate Fungal Cutinases and Initiate the Formation of Infection Organs

The cuticle is considered as the major protective barrier that fungi should overcome. During the earliest stages of infection, fungal cutinases secreted from spores landing on the plant cuticle surface release cutin monomers from the cuticle in a spatially localized manner (Köller et al., 1982; Figure 2). Many reports on pathogenic fungi with different life styles have established the importance of the signaling of these released cutin monomers for the continuation and progression of infection, as it leads to the elevated cutinase activity at later stages of the fungal development essential for the cuticle penetration (Woloshuk and Kolattukudy, 1986; Francis et al., 1996; Gilbert et al., 1996). These include *in vitro* studies of a vast range of pathogenic fungi demonstrating that the cutinase activity significantly increases upon the addition of typical cutin monomers into their growing media, mainly C_16_ and C_18_ *n*-aliphatic primary alcohols and 16-hydroxyhexadecanoic acid. Amongst the pathogenic fungi investigated are the hemibiotrophic fungal species of *Fusarium solani* (Purdy and Kolattukudy, 1975; Lin and Kolattukudy, 1978; Woloshuk and Kolattukudy, 1986), *Colletotrichum graminicola* (Pascholati et al., 1993), and *Colletotrichum gloeosporioides* (Wang et al., 2017), and also the necrotrophic fungal species of *B. cinerea* (van der Vlugt-Bergmans et al., 1997), *Ascochyta rabiei* (Tenhaken et al., 1997), *Pyrenopeziza brassicae* (Davies et al., 2000), *S. sclerotiorum* (Bashi et al., 2012), *Venturia inaequalis* (Köller et al., 1991), and *Monilinia fructicola* (Lee et al., 2010). Another set of reports demonstrated that, apart from activating fungal cutinases, released cutin monomers trigger the formation of spore germ tubes and specialized infection organs, such as appressoria. This was demonstrated in the biotrophic fungal pathogens *Erysiphe graminis* f. sp. *hordei* and hemibiotrophic *Magnaporthe grisea* (Francis et al., 1996; Gilbert et al., 1996; DeZwaan et al., 1999; Zhang et al., 2005).

### The Significant Impact of Cuticle Imperfections on the Nature of Plant–Fungal Interactions

Cutin monomers that are released from the host plant cuticle during infection might shape plant–fungal interactions by endorsing several fungal infection strategies. The question of how defects in cuticle structure, biochemistry, and permeability affect these interactions has puzzled researchers in recent years. Sadler et al. (2016) demonstrated that the physical structure and the precise molecular arrangement of wax molecules affect cuticular permeability, but not the thickness of wax and cutin depositions in the cuticle. In the current section, we elaborate on some of the key reports that inferred the significant impact of cuticle defects on various stages of fungal infection, from the adhesion of fungal spores to the plant surface, through the physical attachment of infection organs, to the capacity of the fungus to penetrate into the inner tissue of the host plant. These studies highlight how these defects might consequently lead to immunity or susceptibility of the host plant upon an attack by pathogenic fungi.

In an early study, Sieber et al. (2000) generated Arabidopsis plants with heterologous overexpression of a cell wall-targeted fungal cutinase from *Fusarium oxysporum*. These transgenic plants, termed CUTE, displayed a striking full immunity to *B. cinerea* despite dramatic modifications in their cuticle ultrastructure and enhanced permeability to solutes and strong postgenital organ fusions (Sieber et al., 2000; Chassot et al., 2007). These results paved the way to the notion that the cuticle is a key component of plant–fungal interactions, and that alterations to its structure and permeability might facilitate immunity to invading fungi. Some follow-up studies geared toward characterizing the interactions between cuticle-deficient mutants and pathogenic fungi further strengthened this notion. For example, the Arabidopsis *lacs2* mutant, deficient in the long-chain acyl-CoA synthetase 2 enzyme that catalyzes the synthesis of fatty acyl-CoA intermediates in the cutin pathway and unsubstituted fatty acids in wax biosynthesis, had a fivefold reduction in the total amount of ꞷ-hydroxylated fatty acids and their derivatives as compared to the WT variant. These modifications led to a strong resistance of the *lacs2* mutant plant to the necrotrophic fungi *B. cinerea* and *S. sclerotiorum* (Schnurr et al., 2004; Bessire et al., 2007). In addition, the 70% reduction in the cutin content of the *cyp86a2/att1* mutant, which is deficient in the CYP86A2 P450-dependent monooxygenase that hydroxylates fatty acids, led to enhanced resistance to *B. cinerea* (Xiao et al., 2004). The *Arabidopsis thaliana* ATP-binding cassette (ABC) protein AtABCG32, an ABC transporter localized to the plasma membrane of epidermal cells, was suggested to export cutin precursors from these cells to the surface (Bessire et al., 2011). The mutation of this gene in the corresponding Arabidopsis *pec1/abcg32* mutant also led to resistance to *B. cinerea*. Fully expanded leaves of this mutant featured significantly lower levels of the cutin monomer C_16_ dicarboxylic and ꞷ-hydroxy C_18:2_ acids, apparently leading to a more permeable cuticle (Fabre et al., 2016). Similarly, rice (*Oryza sativa*) plants with silenced or mutated expression of *OsABCG31*, the homolog of the Arabidopsis ABCG32, displayed increased cuticle permeability and were more resistant to *M. oryzae* (Garroum et al., 2016).

Resistant phenotypes to *B. cinerea* were also detected in the Arabidopsis mutant lines *fiddlehead* (*kcs10*/*fdh*), *lacerata* (*cyp86a8/lcr*), and *bodyguard* (*bdg*), which surprisingly accumulate more cutin, even though they carry mutations in key cuticle biosynthetic genes (Voisin et al., 2009). *kcs10*/*fdh* is deficient in the 3-ketoacyl-CoA synthase 10 condensing enzyme that is part of the fatty acid elongation complex involved in the synthesis of VLCFAs, though its exact function in cuticle formation has yet to be determined (Lolle et al., 1992, 1997; Pruitt et al., 2000); *cyp86a8/lcr* is mutated in CYP86A8, which is involved in the fatty acid hydroxylation pathway (Wellesen et al., 2001); and *bdg* has a mutation in BODYGUARD, an extracellular α/β hydrolase suggested to be involved in cutin polyester assembly (Kurdyukov et al., 2006b; Jakobson et al., 2016).

However, not all plants that feature an increase in cuticle permeability display heightened resistance against pathogenic fungi. The Arabidopsis *hothead* (*hth*) mutant is deficient in its ability to oxidize long-chain ꞷ-hydroxy fatty acids to ꞷ-oxo fatty acids and, therefore, has less α,ꞷ-dicaroxylic fatty acids and more ꞷ-hydroxy fatty acids. This results in a disordered cuticle membrane structure and increased leaf cuticle permeability (Lolle et al., 1998; Kurdyukov et al., 2006a). Conversely, *hth* does not exhibit increased resistance to *B. cinerea* (Bessire et al., 2007). The Arabidopsis double mutant *gpat4/gpat8*, which features altered expression of two glycerol-3-phosphate *sn*-2-acetyltransferases essential for cuticle assembly, is more susceptible to infection by the necrotrophic fungus *A. brassicicola* (Li et al., 2007). Moreover, Arabidopsis mutants that are defective in acyl carrier protein4 (ACP4) and exhibit malformed leaf cuticle are also more susceptible to *B. cinerea* (Xia et al., 2009). Additional studies showed that mutations in SHINE1, a transcription factor of the ethylene response factor (ERF) family that regulates cutin biosynthesis, produce less cutin, and are more susceptible to *B. cinerea* (Kannangara et al., 2007; Sela et al., 2013). Likewise, lower cutin content in the cuticles of the tomato (*Solanum lycopersicum*) fruit skin due to reduced expression of cutin regulator SHINE3 leads to higher susceptibility to *B. cinerea* (Buxdorf et al., 2014). Finally, the cutin polymerization in the tomato fruit skin occurs *via* the transesterification of hydroxyacylglycerol precursors catalyzed by the Gly-Asp-Ser-Leu (GDSL)-motif lipase/hydrolase family protein cutin deficient1 (CD1; Yeats et al., 2012). The fruit skin cuticle of its corresponding mutant, *cd1*, has significantly less cutin, and its fruits are more susceptible to *B. cinerea* (Isaacson et al., 2009).

### Possible Mechanisms by Which Host Plants Perceive Cutin Monomers and/or Cuticle Defects to Elicit Defense Responses

The impressive studies described above provide solid lines of evidence that mutants and transgenic lines with altered cuticular structure and increased permeability exhibit higher resistance to attacks by pathogenic fungi. This concept is still under debate, as other permeable cuticle mutants display an opposite trend, that is, heightened susceptibility to pathogenic fungi. In this section, we mention some of the possible mechanisms that have been raised to explain how host plants perceive cutin monomers and/or changes in cuticle structure and permeability.

Earlier studies showed that the ectopic supplementation of synthetic analogs of typical cutin monomers can confer treated plants with higher resistance against attack by pathogenic fungi. For instance, Namai et al. (1993) treated *Sasanishiki* rice plants with C_18_ epoxy fatty acids and examined the ability of these compounds to inhibit the germination and germ tube elongation of the spores of the rice blast fungus *Pyricularia oryzae*. The authors were able to show that the rate of necrotic lesions formed on the treated leaves was significantly lower than leaves of nontreated control plants, indicating the induction of resistance to the pathogen by the epoxides in the plants. Additionally, uptake experiments using [1-^14^C] derivatives validated that the supplemented epoxides were incorporated into the treated leaves (Namai et al., 1993). In the same way, the topical spray application of synthetic cutin monomers or of a cutin hydrolysate from apple fruit partially protected barley and rice leaves from infection by the fungal pathogens *E. graminis* f.sp. *tritici* and *M. grisea*, respectively. It was further demonstrated that *cis*-9,10-epoxy-18-OH stearic acid (HESA), the most abundant cutin monomer in barley, was amongst the most active compounds. Interestingly, these substances did not seem to have any inhibitory effect on pathogenic fungi when added to their growing media, further suggesting that the resistance observed in these treated plants is associated with the induction of plant defense responses (Schweizer et al., 1994, 1996b). The hypothesis that free cutin monomers are perceived by plant cells as endogenous stress-associated signals were examined in a model system consisting of cultured potato (*Solanum tuberosum*) cells, where, again, HESA was the most active compound in the induction of transient alkalinization of the culture medium, implying an induced defense response. The authors also demonstrated that the application of cutin monomers stimulated the production of the plant stress hormone ethylene and activated the expression of defense-associated genes such as phenylalanine ammonia-lyase (PAL), glutathione S-transferase (GST), and 3-hydroxy-3-methylglutaryl-coenzyme A reductase (HMGR; Schweizer et al., 1996a). Finally, Fauth et al. (1998) showed that adding alkaline hydrolysates of cutin from cucumber (*Cucumber sativus*), tomato, and apple to the epidermal surface of gently abraded hypocotyls of etiolated cucumber seedlings resulted in the generation of H_2_O_2_. The authors concluded that the physiological significance of this might be that upon cuticle degradation by fungal cutinases, the cutin monomers may act as H_2_O_2_ elicitors to induce defense responses (Fauth et al., 1998). Altogether, these reports provide circumstantial evidence that free cutin monomers can be perceived by the host plant cells as chemical signals and endogenous elicitors of defense responses, though the mechanism by which the host plants sense cutin monomers and/or cuticle defects is yet to be fully determined.

Another mechanism raised to explain the link between a permeable cuticle and increased resistance of the host plants to attack by pathogenic fungi involves the accumulation of reactive oxygen species (ROS). A study performed by L’Haridon et al. (2011) proposed that the production of ROS like H_2_O_2_ and O_2_^−^, a permeable cuticle, and increased resistance to invading fungi are all tightly associated. The authors demonstrated that Arabidopsis plants with wounded leaves, plants treated with cutinase, and the cuticle-deficient mutants *bdg* and *lacs2.3*, all produce more ROS and exhibit increased resistance to *B. cinerea*. Remarkably, the authors were able to show that the ROS accumulation and induced resistance occurs under certain conditions only once the cuticle has been permeabilized, and that invading fungi circumvent this mechanism by generating effectors that interfere with the ROS production (L’Haridon et al., 2011). A follow-up study demonstrated that the soft mechanical stress applied to Arabidopsis leaf surfaces by gentle sweeping results in altered cuticle permeability, accompanied by strong resistance to *B. cinerea*, rapid changes in calcium concentrations, and the release of ROS. The authors concluded that Arabidopsis plants can convert gentle forms of mechanical stimuli into strong activation of defense mechanisms against *B. cinerea* (Benikhlef et al., 2013). Finally, the overexpression of DEWAX, an AP2/ERF-type transcription factor that negatively regulates cuticular wax biosynthesis, increases cuticle permeability (Ju et al., 2017). Even though this phenomenon has been attributed more to pronounced changes in cuticular wax deposition than to cutin deposition, both an *in situ* assay of hydrogen peroxide and fluorometric measurements showed that the levels of ROS are higher in DEWAX-overexpressing leaves as compared to the WT leaves. These plants displayed more tolerance to *B. cinerea* infection, accompanied by the upregulation of defense-related genes. Thus, the authors concluded that the increased ROS accumulation and DEWAX-mediated upregulation of defense-related genes are closely associated with enhanced resistance to *B. cinerea* (Ju et al., 2017). Unlike these studies, Dubey et al. (2020) found no difference in ROS levels between cotyledons of WT- and polyunsaturated fatty acid (PUFA)-deficient mutant *fad2-3* Arabidopsis plants, even though this mutant was characterized by cuticle permeability defects (Dubey et al., 2020). To summarize, the above studies suggest an exciting explanation for the increased resistance to *B. cinerea* of several mutants and transgenic plants with a more permeable cuticle and higher ROS production, yet the exact association between these two parameters is not fully understood and requires further examination.

An additional option by which a more permeable cuticle confers resistance relates to the production of fungitoxic substances on the cuticle surface. In fact, fungitoxic activity was measured in diffusates isolated from leaves of the cuticle-deficient mutants *lcr*, *lacs2*, and *pec1/abcg32*, and also the cutinase-expressing CUTE plants (Bessire et al., 2007, 2011; Chassot et al., 2007). Even though it was assumed that fungitoxic activity is associated with the same compound/s in all these cases, the chemical nature of such metabolite/s was not reported. A candidate for such a metabolite was recently raised by Dubey et al. (2020), who identified the over accumulation of 7-methylsulfonylheptyl glucosinolate (7MSOHG) at the cuticle surfaces of (PUFA)-deficient Arabidopsis mutants. Cuticle permeability defects accompanied by arrested hyphal growth were detected in *fad2-1* and *fad* triple mutants of *B. cinerea*. Therefore, the authors linked the appearance of 7MSOHG to defects in cuticle composition and permeability, and resistance to fungi (Dubey et al., 2020). Based on these results, Dubey et al. (2021) investigated the fungi-toxic activity of natural isothiocyanate derivatives of glucosinolates together with semisynthetic glucosinolates and chemical fungicides. The study confirmed that 13 out of the 31 tested were efficient fungicides when applied alone, whereas some operated in a synergistic manner when used in combination against three plant pathogenic fungal species, *Alternaria radicina*, *Fusarium graminearum*, and *Plectosphaerella cucumerina* (Dubey et al., 2021). Altogether, it is reasonable to assume that not only glucosinolates but also fungitoxic metabolites from different biochemical groups play important defensive roles against pathogenic fungi and accumulate at the cuticle surface.

Lastly, defense-related transcriptional responses seem to be common amongst some of the permeable cuticle mutants, raising the possibility that these changes indirectly affect plant–pathogen interactions by conferring resistance against fungi and mounting systemic acquired resistance (SAR). Voisin et al. (2009) compared gene expression changes in young rosette leaves of *lcr*, *fdh*, and *bdg* mutants to that of WT leaves and found commonly upregulated genes that participate in the cuticle and cell wall remodeling and in defense responses upon abiotic stresses and pathogens. Hence, the increased resistant phenotype of these three cuticle-deficient mutants to *B. cinerea* might be the result of primed defense mechanisms that arise due to cuticular defects. To gain deeper insight into the core mechanism by which cuticular defects trigger these types of transcriptional responses, the authors performed an overlap meta-analysis of differentially expressed genes. Using this approach, the *SERRATE* (*SE*) gene was identified and shown to encode a nuclear protein of multiprotein RNA-processing complexes and to be epistatic to *lcr* and *bdg* (Voisin et al., 2009). A link between cuticular defects and defense mechanisms was also proposed for the Arabidopsis *acp4* mutants mentioned earlier. These mutants successfully generated the mobile signal, yet failed to induce SAR. It was demonstrated that the inactivation of SAR is associated with cuticle impairment in these mutants, rather than with alterations in the signaling pathways of the stress-related hormones salicylic and jasmonic acids (Xia et al., 2009).

## Concluding Remarks

In this review, we delineate the multifaceted roles played by epicuticular waxes and released cutin monomers as chemical signaling molecules in the interactions between host plants and pathogenic fungi. The early and recent key reports we present in this fascinating field of research accentuate how these interactions are presumably far more complex than currently assumed. It is evident that these interactions are multifactorial, are regulated simultaneously by many components derived from both the pathogenic fungi and the host plant, and are highly influenced by the biochemical, structural, and permeability properties of the cuticle. Evidence shows that certain wax components affect pre-penetration and infection processes of fungi with different life styles, yet the mechanisms underlying these types of relationships are not fully known. Efforts to elucidate the roles of epicuticular waxes in plant–fungal interactions have thus far mostly utilized mutants with altered wax compositions. However, this approach is still challenging, as in most cases, compositional changes in one biochemical group of wax compounds are typically accompanied by changes in other groups of compounds.

How the cutin monomers released from the cuticle by fungal cutinases during the early stages of infection are recognized by the host plant to elicit defense responses and acquired resistance to pathogens remains a question to be explicated. Thus far, several possible mechanisms have been proposed involving the production of ROS, the accumulation of fungitoxic compounds at the cuticle surface and a primed defense-related transcriptional response, all of which were associated with cuticle defects. In their review, Serrano et al. (2014) suggested that a more permeable cuticle might facilitate the faster perception of signals derived from the cuticle that is being degraded by fungal cutinase during infection and/or that cutin monomers over accumulate in cuticle-deficient mutants due to incomplete cutin polymer assembly. The validity and nature of all these possible mechanisms would require further attention from the research community investigating the field of plant cuticle–pathogenic fungi interactions.

While the cuticle has been solely attributed to aboveground tissues, a recent pioneering study showed that a cuticle-like cell wall structure covers plant root caps and contributes to its protection against abiotic stresses (Berhin et al., 2019). The authors were able to demonstrate that this specialized polyester-rich cuticle is formed in early developing root caps of primary and lateral roots and lost upon the removal of the first root cap cell layer (Berhin et al., 2019). The discovery of cuticle in roots opens a whole new element in the research field of plant cuticle–pathogen interactions, which is of great significance due to the devastating diseases originating from soil-grown pathogenic fungi that attack root tissues. The varied subset of cuticle mutants available today offers an excellent platform with which to examine the possible interactions between root cap cuticles and pathogenic fungi. All in all, the outcome of such efforts is expected to aid the agricultural community to minimize economic and yield losses.

## Author Contributions

GCA, EM, SS, and HC drafted the manuscript. GCA and HC prepared the figures. AA and HC edited the manuscript. All authors contributed to the article and approved the submitted version.

### Conflict of Interest

The authors declare that the research was conducted in the absence of any commercial or financial relationships that could be construed as a potential conflict of interest.
